# The prognostic value of albumin-corrected anion gap for major adverse cardiac events in chronic kidney disease patients undergoing percutaneous coronary intervention

**DOI:** 10.3389/fcvm.2026.1675923

**Published:** 2026-03-27

**Authors:** Ziqiang Guo, Xue Zhang, Yahui Lu, Kangyin Chen

**Affiliations:** 1 Department of Cardiology, Second Hospital of Tianjin Medical University, Tianjin, China; 2 Tianjin Key Laboratory of Ionic-Molecular Function of Cardiovascular Disease, Department of Cardiology, Tianjin Institute of Cardiology, The Second Hospital of Tianjin Medical University, Tianjin, China

**Keywords:** acidosis and oxidative stress, albumin-corrected anion gap (ACAG), chronic kidney disease, coronary artery disease, percutaneous coronary intervention

## Abstract

**Background:**

Cardiovascular disease currently holds the highest morbidity and mortality rates globally. The survival of patients with coronary artery disease (CAD) complicated by chronic kidney disease (CKD) remains a significant threat, posing challenges in management and timely treatment. This study aims to explore the relationship between the Albumin-Corrected Anion Gap (ACAG) and the prognosis of patients with CKD Receiving Percutaneous Coronary Intervention.

**Methods:**

This was a single-center, retrospective study including 973 patients who underwent percutaneous coronary intervention (PCI) at Tianjin Medical University Second Hospital from January 2019 to June 2023, all with an estimated glomerular filtration rate (eGFR) of less than or equal to 60 mL/min/1.73 m^2^. Follow-up was completed in June 2023. The primary efficacy endpoint was the time-to-first occurrence of major adverse cardiovascular events (MACE), defined as the composite of cardiovascular death, recurrent myocardial infarction (MI), or non-fatal stroke. The key secondary endpoint was all-cause mortality, Additional secondary endpoints included (1) the individual components of the primary composite, (2) recurrent MACE (i.e., events after the first occurrence), and (3) any repeat revascularization.

**Results:**

After a follow-up of 918.0 ± 364.7 days, 205 MACEs were recorded. Receiver Operating Characteristic (ROC) curve analysis identified the optimal cutoff value for trial subjects. Compared to the low-ACAG group, the high-ACAG group exhibited a higher incidence of MACEs (29.65% vs. 15.78%, *P* < 0.001) and all-cause mortality (29.92% vs. 11.96%, *P* < 0.001). The Kaplan–Meier survival curve indicated that the low-ACAG group had a higher survival rate. Restricted cubic spline (RCS) analysis suggested that ACAG was positively correlated with MACE events. Univariate and multivariate Cox regression analysis indicated that a high ACAG level (HR: 1.820;95%CI: 1.300–2.546, *P* < 0.001) and the use of diuretics during hospitalization (HR: 1.653;95%CI: 1.195–2.286, *P* = 0.002) were independently associated with the occurrence of MACEs in patients with CAD and CKD. After adding ACAG and eGFR to the traditional risk model, Decision Curve Analysis (DCA) pointed out that DCA could improve the clinical net benefit within a certain range.

**Conclusions:**

Among patients undergoing PCI treatment for chronic kidney disease, elevated ACAG levels are closely related to the patient's cardiac mortality and all-cause mortality rates. Additionally, the use of diuretics in patients with CAD and CKD requires caution and should optimize treatment strategies.

## 1 Introduction

Cardiovascular disease is currently the leading cause of morbidity and mortality worldwide (1). Among them, coronary artery disease (CAD) is the most common disease within the cardiovascular diseases. Statistics show that in China, cardiovascular diseases are one of the main causes of all-cause mortality and disability-adjusted life years (DALYs), resulting in 5.1 million deaths (95% UI: 4.3–5.9) and 100.2 million DALYs in 2021 (2). This burden is particularly significant in the elderly (3) and in special populations with comorbid chronic kidney disease (4). Although, with the improvement of current medical conditions, the survival rate of patients with cardio-renal disease has improved, but this group's survival is still under great threat (5). Therefore, for this group of people, early identification and timely and accurate intervention and treatment are particularly crucial. However, due to the progression of cardio-renal disease and the complex pathological processes involved, this becomes very challenging.

In patients with comorbid chronic kidney disease, due to the gradual loss of residual renal function, the ability to balance acid-base in the body is gradually lost (6). According to the previous study, metabolic acidosis occurs when the GFR decreases to less than 40–50 mL/min/1.73 m^2^ (CKD stage G3) (7), which is associated with an increased risk of MACE+—an association believed to be mediated by systemic inflammation, RAAS activation, endothelial dysfunction, and reduced adiponectin levels (8). The anion gap (AG) represents the difference between unmeasured anions and unmeasured cations in the plasma and is an important indicator for determining the type of metabolic acidosis. However, it is often significantly affected by hypoalbuminemia, which greatly limits its application. The emergence of the albumin-corrected anion gap (ACAG) has largely compensated for the aforementioned shortcomings. ACAG has great value in auxiliary diagnosis, prognosis assessment, and guiding treatment. In addition to cardiovascular diseases, it has been found that ACAG can accurately evaluate the prognosis of patients with kidney disease hypertension (9), pancreatitis (10), ischemic stroke (11), and liver cirrhosis complicated by sepsis (12). However, there are currently no reports on the relationship between ACAG and the prognosis of patients with coronary artery disease and chronic kidney disease. Therefore, we collected data from patients with coronary artery disease and chronic kidney disease at Tianjin Medical University Second Hospital over the past four years, attempting to discover the value of ACAG for the prognosis of this group of patients.

## 2 Methods

### 2.1 Study population

This study included a total of 973 patients who underwent percutaneous coronary intervention (PCI) at Tianjin Medical University Second Hospital from January 2019 to June 2023, and all participants had an estimated glomerular filtration rate (eGFR) of less than or equal to 60 mL/min/1.73 m^2^ (13). PCI patients all had clear symptoms of angina pectoralis and coronary angiography indicated severe coronary stenosis. CKD was defined as eGFR less than or equal to 60 mL/min/1.73 m^2^, derived from the Modification of Diet in Renal Disease (MDRD) formula. All patients included in this study had complete demographic, clinical laboratory, and postoperative follow-up results.

In this study, the primary efficacy endpoint was the time-to-first occurrence of major adverse cardiovascular events (MACE), defined as the composite of cardiovascular death, recurrent myocardial infarction (MI), or non-fatal stroke. The key secondary endpoint was all-cause mortality, Additional secondary endpoints included (1) the individual components of the primary composite, (2) recurrent MACE (i.e., events after the first occurrence), and (3) any repeat revascularization. The exclusion criteria for the study were: 1. Missing relevant laboratory test results, 2. active neoplastic or paraneoplastic syndrome.

### 2.2 Clinical and laboratory data

The electronic medical record system collected data on demographic characteristics and laboratory test results. The gaps in the medical records were obtained by questioning the patients upon admission, including gender, age, past medical history, smoking and drinking history, and related physical examination results. Within 2 h of admission, the first venous blood sample and complete blood cell count results were obtained from all hospitalized patients. ACAG was mainly calculated based on the anion gap and serum albumin results, with the specific formula: ACAG = AG + [44 − Albumin (g/L)] × 0.25 (14). Subsequently, the optimal cutoff value was calculated based on the receiver operating characteristic (ROC) curve analysis and Youden index, and the selected patients were divided into two groups according to the cutoff value. Cardiac ultrasound data were all obtained by experienced sonographers using the PHILIPS iE3 ultrasound diagnostic device. Coronary angiography results were determined by three independent cardiologists evaluating the angiographic impact.

### 2.3 Statistics and analysis

As described above, patients meeting the inclusion criteria were divided into two groups based on the Youden index, namely the low-ACAG group and high-ACAG group. For continuous variables, the mean ± standard deviation (SD) or median (interquartile range) were used for description, depending on the distribution of the data. Categorical variables were represented as frequencies and percentages. For comparisons between the two groups, continuous variables were compared using the t-test (normal distribution) or Mann–Whitney U-test (non-normal distribution), and categorical variables were compared using the *χ*^2^ test or Fisher's exact test. The diagnostic efficacy of the predictive model was assessed using the receiver operating characteristic (ROC) curve, and the predictive ability of the model was quantified by calculating the area under the curve (AUC). Cox proportional hazards regression models were used to evaluate the predictive value of ACAG and other related covariates. Restricted cubic spline (RCS) analysis was used to assess whether there was a nonlinear relationship between ACAG and the main outcomes, and further interaction analysis was applied to assess whether there was any synergistic effect between various factors and the outcomes. To determine whether adding ACAG to the traditional risk model, including other related risk factors (age, diabetes mellitus, Poly-vascular disease, eGFR, the use of diuretics during hospitalization, LVEF), could produce higher clinical net benefit, decision curve analysis (DCA) was employed. In all statistical analyses, a two-tailed *P* < 0.05 was considered significant. All statistical results were analyzed using SPSS statistical software (SPSS 27.0), R 4.4.2, and GraphPad Prism 10.2.3.

## 3 Result

### 3.1 Baseline characteristics

**Figure 1:**
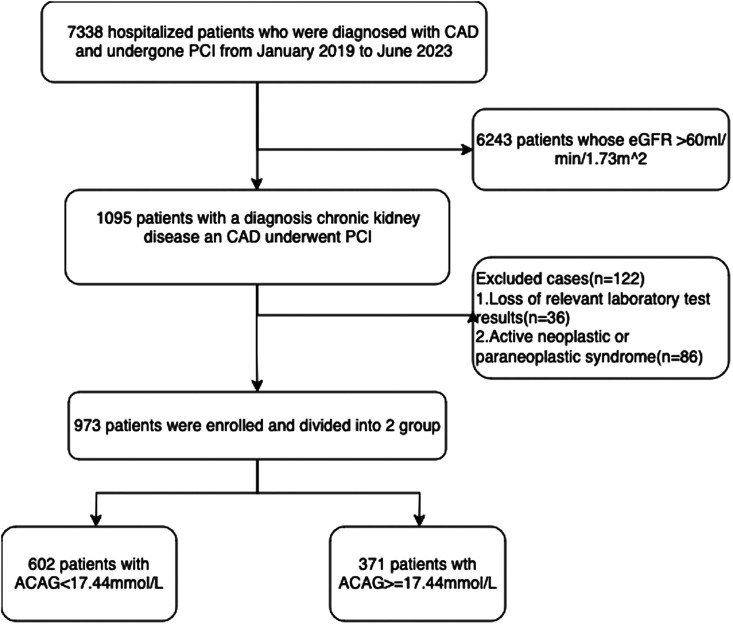
Flowchart of the study cohort.

**Figure 2:**
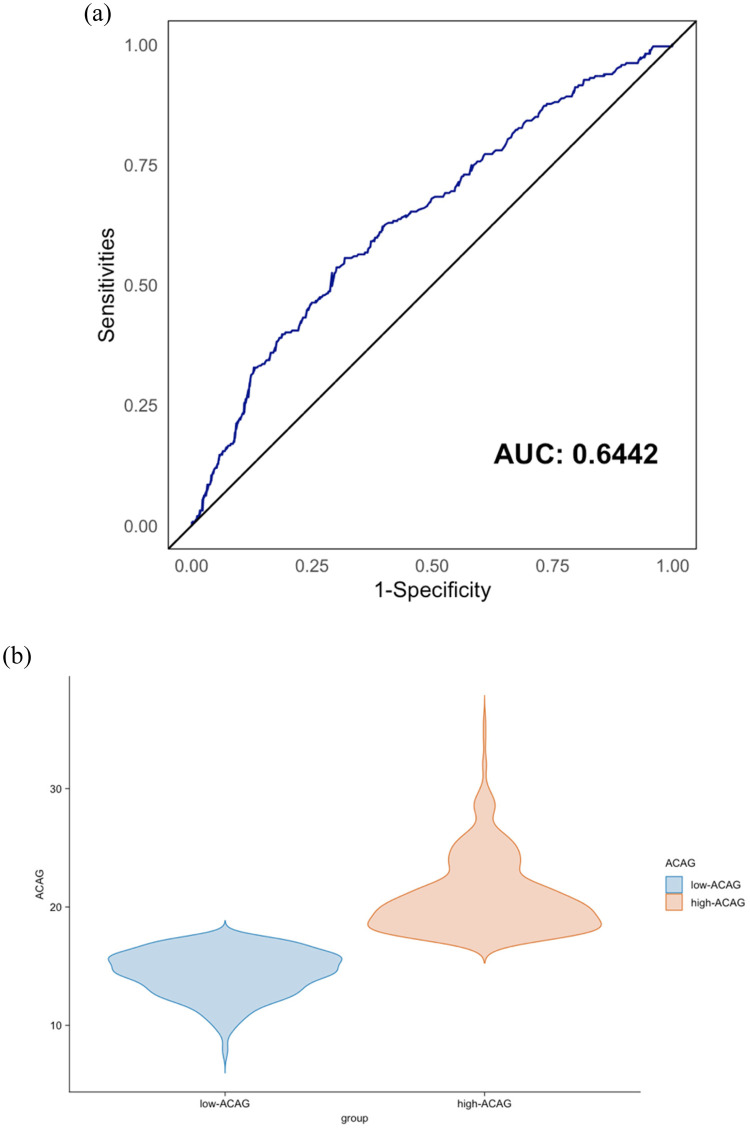
**(a)** Receiver operating characteristic curve (ROC) analysis with the area under the curve of albumin-corrected anion gap (ACAG) in predicting major cardiovascular adverse events (MACEs). **(b)** Different group of ACAG.

As illustrated in Figure 1, after a follow-up period of 918.0 ± 364.7 days, a total of 973 patients diagnosed with coronary artery disease complicated by chronic kidney disease were included in this study. The median age of the patients was 69.26 ± 11.12 years, with 584 males (60.2%) and 389 females (39.8%). As shown in Figure 2a, after determining the major adverse cardiovascular events (MACEs), which included cardiac death, nonfatal myocardial infarction, and nonfatal stroke, the ROC curve analysis identified the optimal cutoff value of the Albumin-Corrected Anion Gap (ACAG) as 17.44 mmol/L (Figure 2a). Based on the derived optimal cutoff value of ACAG, we divided the study population into two groups. Figure 2b shows the difference in baseline ACAG values between the two groups. Table 1 presents the demographic and clinical characteristics of patients in the low-ACAG and high-ACAG groups. Notably, there were no significant differences in gender composition between the two groups, while patients in the high-ACAG group were younger, had lower systolic blood pressure, and faster heart rate upon admission compared to those in the low-ACAG group. Additionally, there were no significant differences in medical history and smoking and drinking history between the two groups. In terms of medication history, the high-ACAG group had fewer patients using Calcium channel blockers, ACE inhibitors or ARBs, Statins, and Glucose-lowering drugs.

**Table 1:** Clinical baseline characteristics of patients according to different groups of ACAG.

Variables	Total (*n* = 973)	Low-ACAG (*n* = 602)	High-ACAG (*n* = 371)	*P*
Age (yrs)	69.26 ± 11.12	69.86 ± 10.80	68.30 ± 11.56	0.033
Gender, male, *n* (%)	584 (60.02)	366 (60.80)	218 (58.76)	0.529
Smoking history (%)	333 (34.22)	208 (34.55)	125 (33.69)	0.784
Alcohol consumption (%)	265 (27.24)	170 (28.24)	95 (25.61)	0.370
SBP (mmHg)	141.97 ± 26.28	144.44 ± 24.80	137.94 ± 28.11	<.001
DBP (mmHg)	80.48 ± 14.28	80.89 ± 13.31	79.80 ± 15.74	0.269
HR (bpm)	79.01 ± 17.76	76.61 ± 15.92	82.97 ± 19.82	<.001
Medical History				
Atrial Fibrillation (%)	72 (7.40)	44 (7.31)	28 (7.55)	0.890
Hypertension (%)	868 (89.21)	541 (89.87)	327 (88.14)	0.399
Diabetes mellitus (%)	464 (47.69)	287 (47.67)	177 (47.71)	0.992
Dyslipidemia (%)	102 (10.48)	68 (11.30)	34 (9.16)	0.292
Biomarker and parameters				
White blood cell (10^9^/L)	8.40 ± 3.59	7.78 ± 2.82	9.41 ± 4.41	<.001
Neutrophil (%)	72.82 ± 10.21	71.33 ± 8.94	75.26 ± 11.60	<.001
Lymphocyte (%)	18.51 ± 8.71	19.92 ± 7.84	16.21 ± 9.54	<.001
Hemoglobin (g/L)	116.34 ± 24.14	119.44 ± 23.01	111.28 ± 25.10	<.001
Hematocrit (%)	35.62 ± 7.34	36.21 ± 7.05	34.66 ± 7.71	0.001
RDW (fl)	18.31 ± 11.41	17.24 ± 10.48	20.05 ± 12.60	<.001
Platelet (10^9^/L)	219.77 ± 70.15	216.65 ± 63.69	224.69 ± 79.09	0.105
Mean Platelet Volume (fl)	9.95 ± 1.10	9.95 ± 1.06	9.95 ± 1.16	0.955
BUN (mmol/L)	13.66 ± 7.48	11.09 ± 5.20	17.82 ± 8.66	<.001
Cr (μmol/L)	281.28 ± 412.69	191.01 ± 413.16	427.74 ± 367.92	<.001
eGFR (mL/min/1.73m^2^)	29.58 ± 19.79	33.07 ± 19.89	23.93 ± 18.29	<.001
UA (μmol/L)	434.45 ± 138.25	427.80 ± 121.86	445.22 ± 160.89	0.074
CO2CP (mmol/L)	22.74 ± 4.02	24.10 ± 3.53	20.55 ± 3.80	<.001
Anion Gap (mmol/L)	16.25 ± 3.90	13.99 ± 2.06	19.93 ± 3.32	<.001
Albumin (g/L)	37.41 ± 4.89	38.25 ± 4.85	36.06 ± 4.66	<.001
Total Cholesterol (mmol/L)	4.45 ± 1.25	4.53 ± 1.20	4.32 ± 1.31	0.013
Triglyceride (mmol/L)	1.76 ± 1.08	1.76 ± 1.10	1.76 ± 1.04	0.927
HDL-c (mmol/L)	1.01 ± 0.29	1.05 ± 0.29	0.95 ± 0.28	<.001
LDL-c (mmol/L)	2.84 ± 1.00	2.88 ± 0.96	2.77 ± 1.05	0.091
VLDL-c (mmol/L)	0.60 ± 0.33	0.60 ± 0.34	0.61 ± 0.31	0.744
Fast blood Glucose (mmol/L)	9.11 ± 4.91	8.74 ± 4.14	9.70 ± 5.90	0.007
LVEF (%)	51.30 ± 12.23	53.20 ± 11.83	48.22 ± 12.24	<.001
Discharge medications				
DAPT	838 (86.13)	333 (89.76)	505 (83.89)	<.010
ARNI (%)	358 (36.79)	216 (35.88)	142 (38.27)	0.452
Beta-blocker (%)	554 (56.94)	339 (56.31)	215 (57.95)	0.616
Calcium channel blocker (%)	457 (46.97)	311 (51.66)	146 (39.35)	<.001
ACEi or ARB (%)	133 (13.67)	111 (18.44)	22 (5.93)	<.001
Diuretics (%)	429 (44.09)	256 (42.52)	173 (46.63)	0.210
Statins (%)	854 (87.77)	542 (90.03)	312 (84.10)	0.006
Glucose-lowering drugs (%)	288 (29.60)	197 (32.72)	91 (24.53)	0.007
Diseased coronary artery				
LM (%)	103 (10.59)	63 (10.47)	40 (10.78)	0.876
Multi-vessel disease (%)	882 (90.65)	541 (89.87)	341 (91.91)	0.287

SBP, systolic blood pressure; DBP, diastolic blood pressure; HR, heart rhythm; RDW, red cell distribution width; BUN, blood urea nitrogen; Cr, creatinine; eGFR, estimated glomerular filtration rate; UA, uric acid; CO2CP, carbon dioxide combining power; HDL-C, high density lipoprotein cholesterol; LDL-C, low density lipoprotein cholesterol; VLDL-c, very low density lipoprotein cholesterol; LVEF, left ventricular ejection fraction; DAPT, dual antiplatelet therapy; ARNI, angiotensin receptor-neprilysin inhibitors; ACEI, angiotensin-converting enzyme inhibitor; ARB, angiotensin receptor blocker; LM, left main coronary artery disease.

In the collected clinical laboratory and examination data, patients in the high-ACAG group had significantly higher levels of white blood cells, neutrophil ratio, red blood cell distribution width, blood urea nitrogen, blood creatinine, anion gap, and fasting blood glucose compared to those in the low-ACAG group; moreover, patients in the low-ACAG group had higher lymphocyte ratio, blood albumin, red blood cell hematocrit, eGFR, carbon dioxide combining capacity, serum albumin, total cholesterol, HDL-c, and LVEF (*P* < 0.05). Apart from the aforementioned indicators, no significant differences were observed in other indicators, and there were no significant differences in the coronary angiography results between the two groups in terms of left main coronary artery disease [63 (10.47%) vs. 40 (10.78%), *P* = 0.786] and multivessel disease [541 (8.97%) vs. 341 (91.91%), *P* = 0.287] proportions.

### 3.2 Association between albumin-corrected anion gap and clinical prognosis

**Figure 3:**
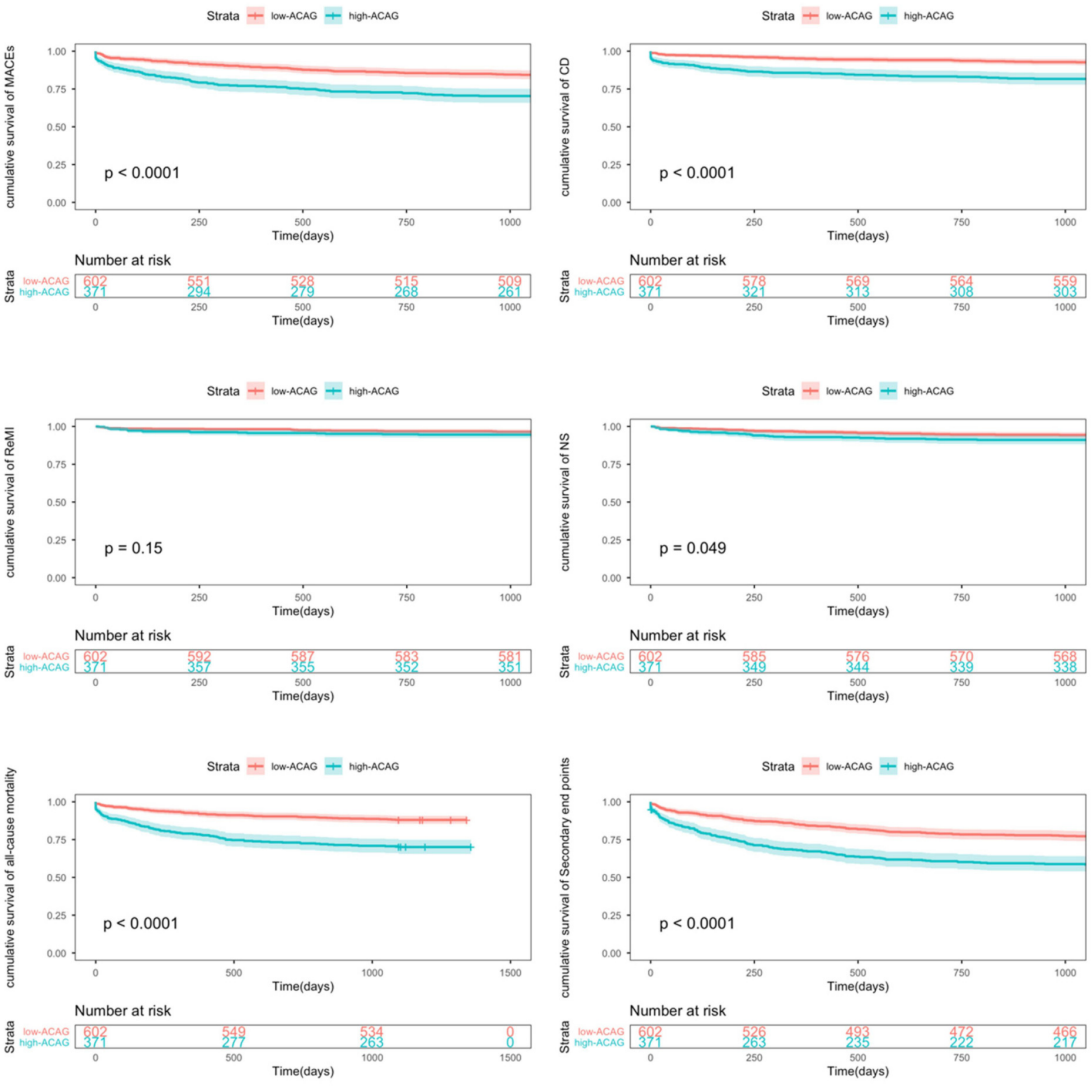
Kaplan–Meier survival curve analysis showing MACEs, cardiac death (CD), recurrent MI, nonfatal stroke (NS), all-cause mortality, secondary end points.

As shown in Table 2, after a follow-up period of 918.0 ± 364.7 days, a total of 119 patients experienced cardiac death, 67 patients had nonfatal strokes, 41 patients suffered recurrent myocardial infarction, 52 patients underwent repeat coronary revascularization, and 293 patients experienced secondary end events. In the high-ACAG group, the incidence of MACEs (Major Adverse Cardiovascular Events), cardiac death (CD), and secondary outcomes was significantly higher than in the low-ACAG group (*P* < 0.001). However, there were no significant differences in the incidence of nonfatal stroke, recurrent myocardial infarction, and repeat coronary revascularization between the two groups. As depicted in Figure 3, the Kaplan–Meier survival curve and Log-rank sum test also revealed that patients in the high-ACAG group had a higher rate of MACEs, CD and all-cause mortality events. In summary, higher ACAG levels were associated with an increased incidence of cardiovascular events.

**Table 2:** Prognosis of patients with different groups of ACAG.

Clinical endpoints	Low-ACAG (*n* = 602)	High-ACAG (*n* = 371)	*P*-value
MACEs	95 (15.78%)	110 (29.65%)	<.001
CD	48 (7.97%)	71 (19.14%)	<.001
NS	34 (5.65%)	33 (8.89%)	0.052
ReMI	21 (3.49%)	20 (5.39%)	0.151
All-cause mortality	72 (11.96%)	111 (29.92%)	<.001
Revascularization (PCI or CABG)	34 (5.65%)	18 (4.85%)	0.637
Secondary end points	140 (23.26%)	153 (41.24%)	<.001

MACE, major adverse cardiovascular events; CD, cardiac death; NS, nonfatal stroke; ReMI, recurrent myocardial infarction; PCI, percutaneous coronary intervention; CABG, coronary artery bypass grafting. Secondary end events include cardiac death, nonfatal myocardial infarction, nonfatal stroke, and revascularization.

### 3.3 RCS analysis of albumin-corrected anion gap and MACEs

**Figure 4:**
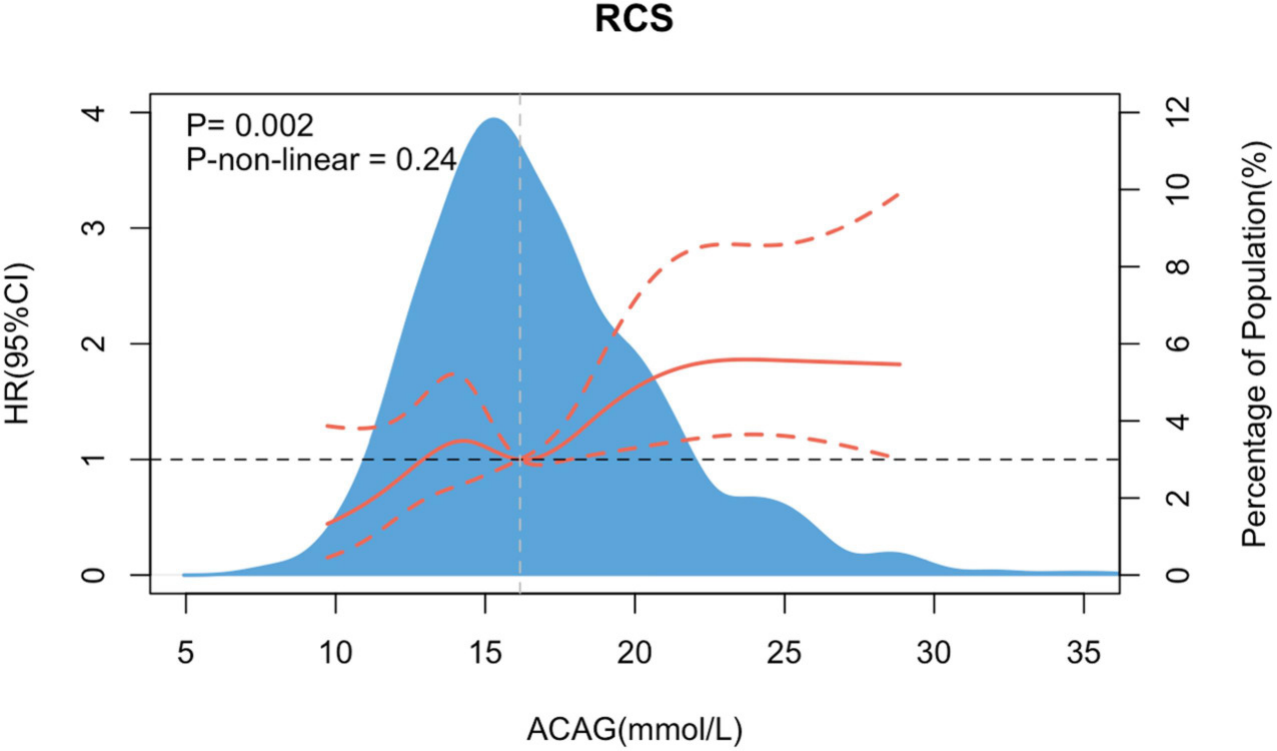
The association between MACEs and ACAG.

After conducting a restricted cubic spline (RCS) analysis, we found that there was no statistically significant nonlinear trend between the Albumin-Corrected Anion Gap (ACAG) and the occurrence of Major Adverse Cardiovascular Events (MACEs) (*P*-non-linear=0.24). As shown in Figure 4, with the increase of ACAG, the incidence of MACEs also showed a significant rise. However, after ACAG increased to a certain level, the rate of MACEs event occurrence tended to decline and maintained at a relatively stable higher risk level.

### 3.4 ACAG was an independent risk factor for prognosis among patients with diagnosed CAD and CKD

As shown in Table 3, to further verify whether there was an independent association between Albumin-Corrected Anion Gap (ACAG) and the occurrence of Major Adverse Cardiovascular Events (MACEs) in patients, we conducted univariate and multivariate Cox regression analyses. According to the results, an ACAG level of ≥17.44 mmol/L (HR: 1.820; 95% CI: 1.300–2.546, *P* < 0.001) was an independent predictive risk factor for MACEs. Additionally, age, diabetes mellitus, Multi-vessel disease, estimated glomerular filtration rate (eGFR), the use of diuretics during hospitalization (HR: 1.653; 95% CI: 1.195–2.286, *P* = 0.002), and left ventricular ejection fraction (LVEF) were also independent predictive risk factors. While DAPT did not qualify as an independent predictive risk factors of prognosis in this cohort, presumably because too few patients were managed without DAPT, leaving the analysis underpowered.

**Table 3:** Cox regression analysis.

Variables	Univariable Cox regression		Multivariable Cox regression	
	HR (95%CI)	*P*	HR (95%CI)	*P*
Gender	0.951 (0.720–1.256)	0.725	1.201 (0.883–1.632)	0.243
Age	1.025 (1.012–1.039)	<0.001	1.033 (1.017–1.050)	<0.001
Smoking history	0.825 (0.613–1.112)	0.206		
Atrial Fibrillation	1.486 (0.946–2.334)	0.086		
Hypertension	1.448 (0.869–2.413)	0.155		
SBP	1.001 (0.996–1.006)	0.683		
DBP	0.987 (0.977–0.997)	0.008	0.992 (0.982–1.003)	0.173
HR	1.000 (0.993–1.008)	0.931		
Dyslipidemia	1.103 (0.834–1.458)	0.492		
Diabetes mellitus	1.509 (1.144–1.990)	0.004	1.433 (1.073–1.913)	0.015
White blood cell	1.040 (1.007–1.075)	0.019	1.010 (0.968–1.054)	0.344
Neutrophil (%)	1.023 (1.009–1.038)	0.001	1.003 (0.987–1.019)	0.707
Hemoglobin	0.987 (0.981–0.993)	<0.001	0.994 (0.987–1.002)	0.117
Platelet	0.998 (0.996–1.000)	0.086		
CO2CP	0.959 (0.926–0.992)	0.016	1.022 (0.981–1.064)	0.282
Triglyceride	0.921 (0.800–1.061)	0.256		
Total Cholesterol	0.994 (0.890–1.111)	0.921		
LDL-c	0.989 (0.861–1.137)	0.881		
HDL-c	1.077 (0.671–1.728)	0.760		
Fast blood Glucose	1.019 (0.994–1.045)	0.141		
UA	1.001 (1.000–1.002)	0.282		
eGFR	0.953 (0.987–1.000)	0.055	0.987 (0.977–0.997)	0.009
BUN	1.033 (1.017–1.049)	<0.001	1.005 (0.981–1.030)	0.682
ACAG				
Q1	Ref		Ref	
Q2	2.084 (1.584–2.743)	<0.001	1.820 (1.300–2.546)	<0.001
LM	0.980 (0.624–1.539)	0.931		
multi-vessel disease	0.364 (0.180–0.738)	0.005	2.525 (1.102–5.787)	0.029
DAPT	2.129 (1.258–3.601)	0.005	1.600 (0.934–2.741)	0.087
statins	0.852 (0.537–1.352)	0.497		
ARB	0.793 (0.544–1.156)	0.228		
ARNI	0.851 (0.636–1.138)	0.277		
ACEi or ARB	0.672 (0.428–1.055)	0.084		
Beta-blocker	1.256 (0.947–1.665)	0.113		
CCBs	1.124 (0.855–1.478)	0.403		
diuretics	2.124 (1.605–2.810)	<0.001	1.653 (1.195–2.286)	0.002
Glucose-lowering drugs	1.097 (0.816–1.474)	0.540		
LVEF	0.974 (0.964–0.984)	<0.001	0.987 (0.975–0.999)	0.033

SBP, systolic blood pressure; DBP, diastolic blood pressure; HR, heart rhythm; CO2CP, carbon dioxide combining power; HDL-C, high density lipoprotein cholesterol; LDL-C, low density lipoprotein cholesterol; UA, uric acid; BUN, blood urea nitrogen; eGFR, estimated glomerular filtration rate; DAPT, dual antiplatelet therapy; ARNI, angiotensin receptor-neprilysin inhibitors; ACEI, angiotensin-converting enzyme inhibitor; ARB, angiotensin receptor blocker; LM, left main coronary artery disease; CCBs, calcium channel blocker; LVEF, left ventricular ejection fraction.

Cox proportional-hazards regression model analyses were performed using four separate models to detect independent predictors of clinical prognosis (Table 4). In Model IV, after adjusting for all relevant confounders except ACAG, a high level of ACAG remained an independent risk factor for increased MACEs events (HR: 1.825; 95% CI: 1.367–2.436; *P* < 0.001), cardiac death events (HR: 2.114; 95% CI: 1.438–3.108; *P* < 0.001), and secondary outcomes (HR: 1.833; 95% CI: 1.441–2.332; *P* < 0.001).

**Table 4:** The association of high ACAG (≥17.44 mmol/L) and future adverse events in patients.

Clinical endpoints	Model I	Model II	Model III	Model IV
Cardiac death	2.584 (1.791–3.727) *P* < 0.001	2.836 (1.962–4.097) *P* < 0.001	2.685 (1.857–3.884) *P* < 0.001	2.114 (1.438–3.108) *P* < 0.001
Nonfatal myocardial infarction	1.563 (0.847–2.884) *P* = 0.153	1.540 (0.834–2.846) *P* = 0.168	1.554 (0.839–2.876) *P* = 0.161	1.438 (0.750–2.760) *P* = 0.274
Nonfatal stroke	1.612 (0.998–2.602) *P* = 0.051	1.609 (0.995–2.602) *P* = 0.052	1.572 (0.971–2.543) *P* = 0.066	1.517 (0.916–2.513) *P* = 0.106
All-cause mortality	1.363 (1.010–1.839) *P* = 0.043	1.502 (1.100–2.051) *P* = 0.01	1.507 (1.106–2.056) *P* = 0.009	1.357 (0.974–1.892) *P* = 0.071
Revascularization (PCI or CABG)	0.861 (0.486–1.525) *P* = 0.609	0.836 (0.471–1.484) *P* = 0.542	0.809 (0.455–1.438) *P* = 0.470	0.817 (0.447–1.493) *P* = 0.512
MACEs	2.084 (1.584–2.743) *P* < 0.001	2.218 (1.683–2.923) *P* < 0.001	2.135 (1.620–2.815) *P* < 0.001	1.825 (1.367–2.436) *P* < 0.001
Secondary end events	2.064 (1.641–2.596) *P* < 0.001	2.136 (1.696–2.690) *P* < 0.001	2.086 (1.656–2.628) *P* < 0.001	1.833 (1.441–2.332) *P* < 0.001

MACEs include cardiovascular death, nonfatal myocardial infarction, and nonfatal stroke. Secondary end events include cardiac death, nonfatal myocardial infarction, nonfatal stroke, and revascularization. Model I: confounding factors were not controlled. Model II: adjusted with age and gender. Model III: adjusted with age, gender, diabetes, the use of diuretics during hospitalization, multi-vessel disease. Model IV: adjusted with age, gender, diabetes, the use of diuretics during hospitalization, multi-vessel disease, LVEF, eGFR.

### 3.5 Subgroup analyses

**Figure 5:**
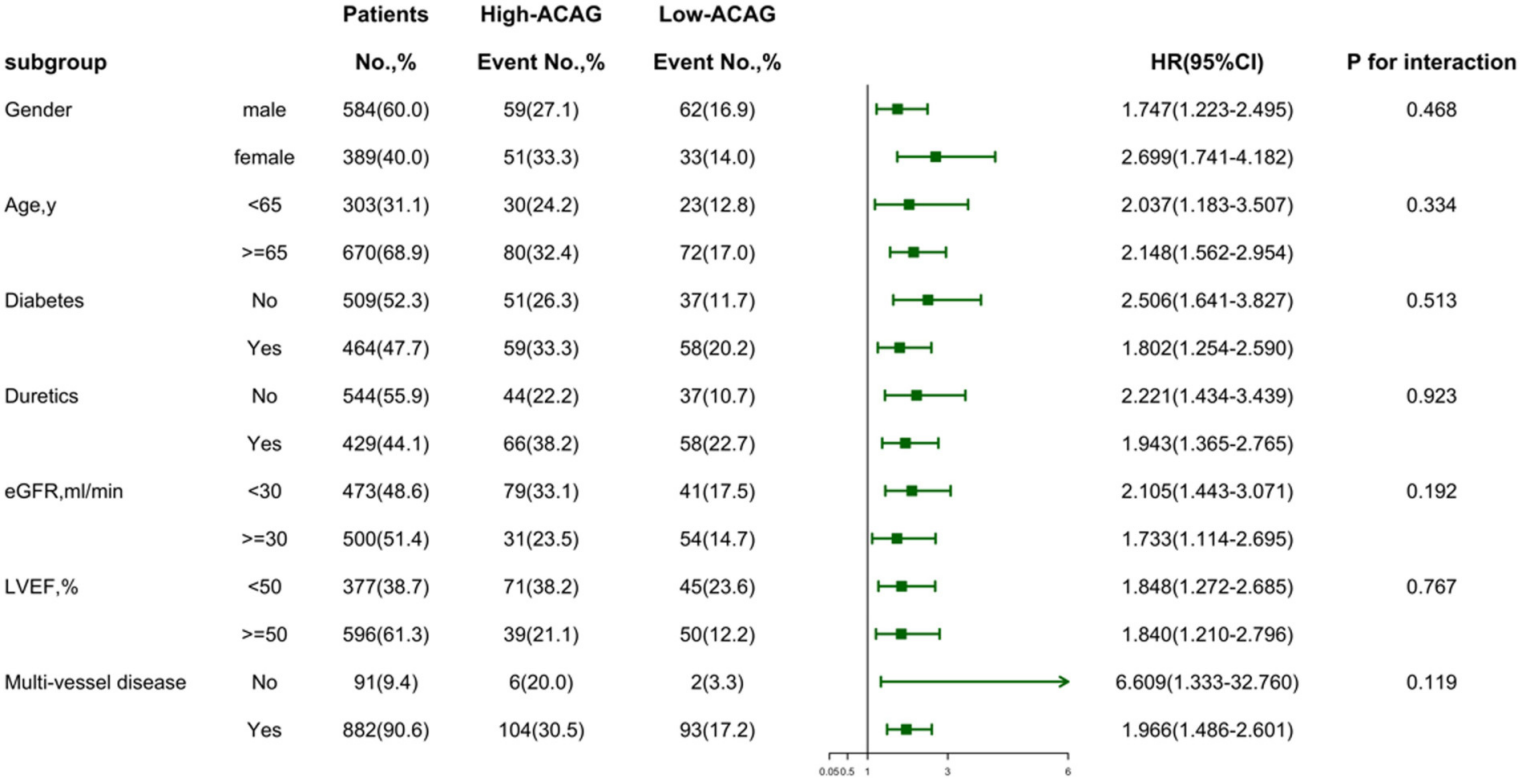
The association of high ACAG (≥17.44 mmol/L) and future adverse events in patients.

As is shown in Figure 5 and Table 5, subgroup analyses were conducted based on age, gender, hypertension, diabetes, newly diagnosed dyslipidemia, smoking, the use of diuretics during hospitalization, DBP on admission, eGFR. The relationship between ACAG and MACEs remained consistent across all subgroups. Additionally, no significant interactions were observed between ACAG and the stratification variables (*P* for interaction >0.05). However, in the analysis of additional interaction statistics, there is potential influence on ACAG between both LVEF (SI: 1.74; 95% CI: 1.07–2.85) and eGFR (SI: 1.91; 95% CI: 1.18–3.09).

**Table 5:** Additive interaction statistics between ACAG and different subgroups.

Subgroup		Patients No., %	RERI	AP	SI
Gender	male	584 (60.0)	−0.82 (−25.07–18.53)	−0.17 (−1.38–0.76)	0.82 (0.49–1.36)
	female	389 (40.0)			
Age, y	<65	303 (31.1)	2.12 (−19.79–36.27)	0.27 (−0.55–0.82)	1.45 (0.85–2.50)
	>=65	670 (68.9)			
Diabetes	No	509 (52.3)	0.91 (−21.60–31.41)	0.13 (−1.00–0.74)	1.19 (0.74–1.89)
	Yes	464 (47.7)			
Diuretics	No	544 (55.9)	2.44 (−18.66–44.24)	0.28 (−0.85–0.76)	1.48 (0.89–2.45)
	Yes	429 (44.1)			
eGFR, mL/min	<30	473 (48.6)	3.55 (−17.07–45.37)	0.38 (−0.34–0.83)	1.74 (1.07–2.85)
	>=30	500 (51.4)			
LVEF, %	<50	377 (38.7)	4.35 (−15.47–51.84)	0.43 (−0.32–0.85)	1.91 (1.18–3.09)
	>=50	596 (61.3)			
Multi-vessel disease	No	91 (9.4)	−1.12 (−35.75–27.51)	−0.14 (−1.47–0.45)	0.87 (0.4–1.87)
	Yes	882 (90.6)			

RERI, relative excess risk due to interaction; AP, attributable proportion due to interaction; SI, synergy index.

### 3.6 Adding ACAG and eGFR to the baseline model had superior clinical value

**Figure 6:**
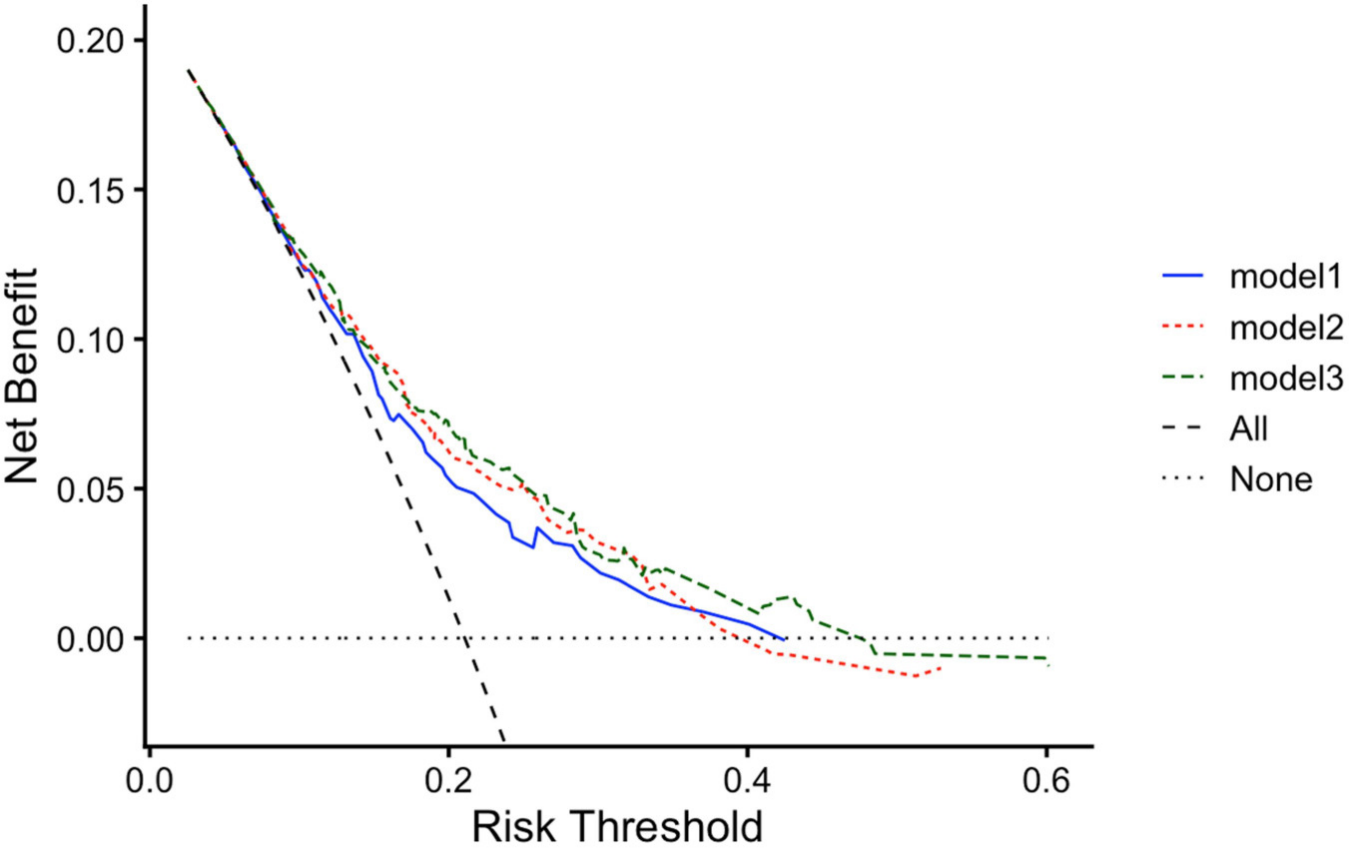
Decision curves analysis (DCA) for model predicting ACAG ≥ 17.44 mmol/L. Model 1: adjusted with age, gender, diabetes, the use of diuretics during hospitalization, LVEF, multi-vessel disease. Model 2: adjusted for Model 1 plus eGFR. Model 3: adjusted for Model 2 plus ACAG.

To further validate the clinical value of ACAG in predicting MACEs, we performed decision curve analysis (DCA) for MACEs events. The results, as shown in Figure 6, indicated that adding ACAG and eGFR to the baseline Model 1 significantly improved the net benefit within a certain probability range. This suggests that adding ACAG and eGFR to the traditional risk model is more helpful for clinical decision-making and provides higher clinical value and benefit when predicting adverse cardiovascular outcomes in patients diagnosed with coronary artery disease (CAD) and chronic kidney disease (CKD).

### 3.7 Sensitivity analysis

To validate the relationship between ACAG and MACEs across different grouping strategies, we subsequently analyzed ACAG as a dichotomized variable (using the optimal cut-off from ROC analysis), quartiles, and a continuous variable. As shown in Supplementary Table S2, all grouping forms demonstrated significant associations with MACEs. In the quartile analysis, compared with Q1 (7.4–14.21 mmol/L, *n* = 243), neither Q2 (14.21–16.15 mmol/L, *n* = 242) nor Q3 (16.15–19.09 mmol/L, *n* = 245) showed statistically significant differences, whereas the highest quartile (Q4: 19.09–35.68 mmol/L, *n* = 243) exhibited a significantly increased risk of MACEs (HR: 2.070, 95% CI: 1.346–3.181, *P* < 0.001). Multivariable Cox regression (Supplementary Table S1) confirmed these findings, with other risk factors (except sex) remaining independent predictors. DCA (Supplementary Figures S1, S3) indicated that dichotomized grouping yielded higher net clinical benefit within certain decision thresholds. Kaplan–Meier curves and log-rank tests (Supplementary Figure S2) further demonstrated elevated rates of MACEs, cardiac death, and all-cause mortality in Q4. Model comparison using AIC and BIC (Supplementary Table S3) favored the cut-off-based model, which showed lower values and a substantially higher optimal frequency. These supplementary analyses reinforce our primary conclusions and support the robustness of our findings.

## 4 Discussion

In this retrospective study, we thoroughly examined the role of the albumin-corrected anion gap (ACAG) in patients suffering from coronary artery disease (CAD) complicated by chronic kidney disease (CKD). Our results uncovered a significant correlation between ACAG and the prognostic prediction in patients with CAD and CKD, providing new insights for clinical diagnosis and treatment decisions. Specifically, through the analysis of 973 patient medical records, we identified a non-linear relationship between ACAG levels and major adverse cardiovascular events (MACEs). This relationship was robustly confirmed using restricted cubic spline (RCS) analysis, Cox regression and other research methods. Unfortunately, within subgroups defined by baseline characteristics, no cardiovascular events were observed among the very small number of patients with poor DAPT compliance, leading to complete separation and precluding inclusion of DAPT compliance status in the multivariate Cox regression analysis. However, this initial finding indicates that ACAG is a potential prognostic marker for adverse cardiovascular events in CAD patients complicated with CKD, deserving further exploration in prospective studies to assess its clinical utility.

As an important indicator for assessing acid-base balance, the Anion Gap (AG) has significant value in diagnosing acidosis, but it is highly susceptible to the influence of serum albumin (15). Patients with hypoalbuminemia may have a falsely reduced AG due to reduced albumin, thereby masking the diagnosis of metabolic acidosis (16). Figge et al. found that for every 10 mg/L decrease in serum albumin, AG decreases by 2.5 mmol/L (14). The introduction of ACAG addresses the impact of changes in serum albumin on AG and can more accurately assess acid-base balance status. Previous studies have revealed that metabolic acidosis in CKD is associated with an increased risk of MACE, as well as its individual components including incident HF, stroke, MI, and CV death (8). However, in our cohort, progressive acidosis (as reflected by higher ACAG levels) was specifically linked to CV death among the MACE components, a discrepancy likely attributable to the limited sample size and the low incidence of MI and stroke in our data.

Additionally, the role of ACAG in cardiovascular and kidney disease outcomes has also been widely discussed in numerous other studies. Sheng et al. (17) and Zhao et al. (18) found that elevated serum ACAG levels were associated with new-onset heart failure and poor prognosis after acute myocardial infarction. In addition, in patients with atrial fibrillation (AF), there is a linear relationship between ACAG levels and all-cause mortality. Studies showed that higher ACAG levels independently predict 30-day and 1-year mortality rates, and this association remains significant even after adjusting for confounding factors (19). In a retrospective study based on MIMIC-IV database, Gao (20) found that high ACAG was significantly associated with 30-day and 360-day in-hospital mortality in AKI patients. A study evaluating the prognostic value of ACAG in patients with cardiogenic shock also found that elevated ACAG levels were significantly associated with the prognosis of cardiogenic shock patients (21). Therefore, elevated ACAG may reflect multi-organ dysfunction in the body, indicating its potential value in assessing disease severity.

Chronic kidney disease has become a significant public health issue globally. As the disease progresses, the risk of cardiovascular diseases in patients with chronic kidney disease also increases (22). In addition to the high prevalence of traditional coronary artery disease (CAD) risk factors such as diabetes and hypertension, patients with CAD are also exposed to uremia-related cardiovascular disease risk factors, including inflammation, oxidative stress, and abnormal calcium-phosphate metabolism (23). The management of CAD in CKD patients is complex (24), and due to the complex pathological processes, it becomes very difficult to grasp the timing of treatment and the choice of drugs (25).Changes in acid-base balance can reflect changes in the circulatory system to some extent and play an important role in maintaining the function of the cardiovascular and renal systems (26). In patients with chronic kidney disease, due to reduced kidney function, which decreases excretion of acidic metabolic products and impairs urinary acidification, metabolic acidosis is prone to occur (27). The albumin-corrected anion gap (ACAG), calculated from routinely available serum electrolytes and albumin, provides a convenient and reliable measure of metabolic acidosis severity.

Our present findings indicate that elevated ACAG levels predict worse clinical outcomes in CKD patients who have undergone percutaneous coronary intervention. Mechanistically, as acidosis worsens, endothelial nitric-oxide synthase (eNOS) activity declines, reducing nitric-oxide (NO) production and blunting endothelium-dependent vasodilation. Concomitantly, the low pH indirectly activates the renin–angiotensin–aldosterone system (RAAS), thereby triggering NF-κB signaling and endoplasmic-reticulum stress that drive endothelial cells toward a pro-inflammatory phenotype (28). Although direct data on in-stent restenosis or neointimal remodeling are still lacking, these converging lines of evidence indicate that acidosis is unlikely to favor optimal vascular healing after percutaneous coronary intervention (PCI), providing a mechanistic basis for the observed association between elevated ACAG levels and worse clinical prognosis.

In addition to the traditional risk factors for coronary artery disease and chronic kidney disease being associated with poor prognosis, medication factors should not be overlooked (29). This study found that the use of diuretics during hospitalization was also associated with poor outcomes in patients with CAD and CKD. This finding may be explained by the fact that our study did not exclude patients with heart failure (LVEF < 50%) and severe kidney dysfunction, and the specific dosage and type of diuretics used were not precisely defined. Nevertheless, currently, when edema occurs in CKD patients, diuretics can alleviate the body's fluid retention by increasing the excretion of sodium and water, improving kidney function. Therefore, diuretics are still a first-line treatment (30). However, after kidney disease patients receive diuretic treatment, especially with high doses, a considerable proportion of the population will experience reduced renal perfusion, neuroendocrine system abnormalities, drug interactions, electrolyte and acid-base disorders, and hypoalbuminemia, and develop diuretic resistance, ultimately increasing the readmission rate and mortality rate (31, 32). As described above, although diuretics play an important role in managing heart failure in CKD patients, their use should be cautious to avoid the deterioration of kidney function and diuretic resistance.

This study has certain limitations. First, this study is a single-center retrospective study with a limited sample size, and selection bias cannot be avoided. Besides, the number of patients with poor DAPT compliance was too small. Further multi-center studies and long-term follow-ups are needed to verify the above results and the influence of poor compliance of DAPT. Second, when building the model, as the adjustment variables were included, the association between ACAG and prognosis showed an attenuating trend, which may be related to different statistical methods or LVEF and eGFR having more significant predictive value. In addition, this study only included the relevant laboratory examination indicators at admission, and failed to dynamically monitor the trajectories of relevant indicators (including eGFR and LVEF). Additionally, the specific dosages and types of diuretics were not precisely documented. Therefore, more detailed tracking and follow-up of the condition, medication history, and laboratory examination of related to the patients are needed. Finally, in this study, the evaluation of multivessel disease was based solely on coronary angiography imaging findings, which may introduce measurement bias. Further proper evaluation should be conducted through syntax scoring and coronary functional examination (such as FFR, iFR, QFR, etc.).

## 5 Conclusion

Among patients with chronic kidney disease undergoing PCI treatment, elevated ACAG levels are closely related to the patients’ cardiac mortality and all-cause mortality rates. In addition, the use of diuretics in patients with chronic kidney disease requires caution and optimized treatment strategies should be applied.

### 5.1 Perspective

In the future, dedicated acid-lowering therapy should be prospectively evaluated in patients with overt metabolic acidosis. In individuals with advanced renal insufficiency, incorporation of the anion-gap–corrected acidosis (ACAG) parameter into the GRACE score for those presenting with NSTE-ACS may further refine risk stratification.

## Data Availability

The datasets presented in this article are not readily available because this study has certain limitations. First, this study is a single-center retrospective study with a limited sample size, and the selection bias cannot be avoided. Further multi-center studies and long-term follow-ups are needed to verify the above results. Second, when building the model, as the adjustment variables were included, there was a trend of ACAG and the relationship with the prognosis decreased, which may be related to different statistical methods or LVEF and eGFR having more significant predictive value. In addition, this study only included the relevant laboratory examination indicators at admission, and failed to dynamically monitor the trajectory of changes in related indicators (including eGFR, LVEF), and the specific dosage and type of diuretics used were not precisely counted, therefore, more detailed tracking and follow-up of the condition, medication history, and laboratory examination of related to the patients are needed. Finally, in this study, the evaluation of multivessel disease was only summarized through the imaging performance of coronary angiography at the time of imaging, which there is a certain measurement bias. Further proper evaluation should be conducted through syntax scoring and coronary functional examination (such as FFR, iFR, QFR, etc.). Requests to access the datasets should be directed to Ziqiang Guo, guoziqbzg@163.com.
