# Supplementary material for: The prognostic value of albumin-corrected anion gap for major adverse cardiac events in chronic kidney disease patients undergoing percutaneous coronary intervention
Source: Front Cardiovasc Med. 2026 Mar 27;13:1675923. doi: 10.3389/fcvm.2026.1675923 (PMC13065508; doi:10.3389/fcvm.2026.1675923)
Supplement: Supplementary file 1 [file Table1.docx]

**Figure S1** Decision Curve Analysis (DCA) for continuous ACAG in predicting MACEs.

**Figure S2** Kaplan-Meier survival curves for clinical outcomes by ACAG quartiles.

**Figure S3** Decision Curve Analysis (DCA) of ACAG quartiles for predicting MACEs.

**Figure S1** Decision Curve Analysis (DCA) for continuous ACAG in predicting MACEs.


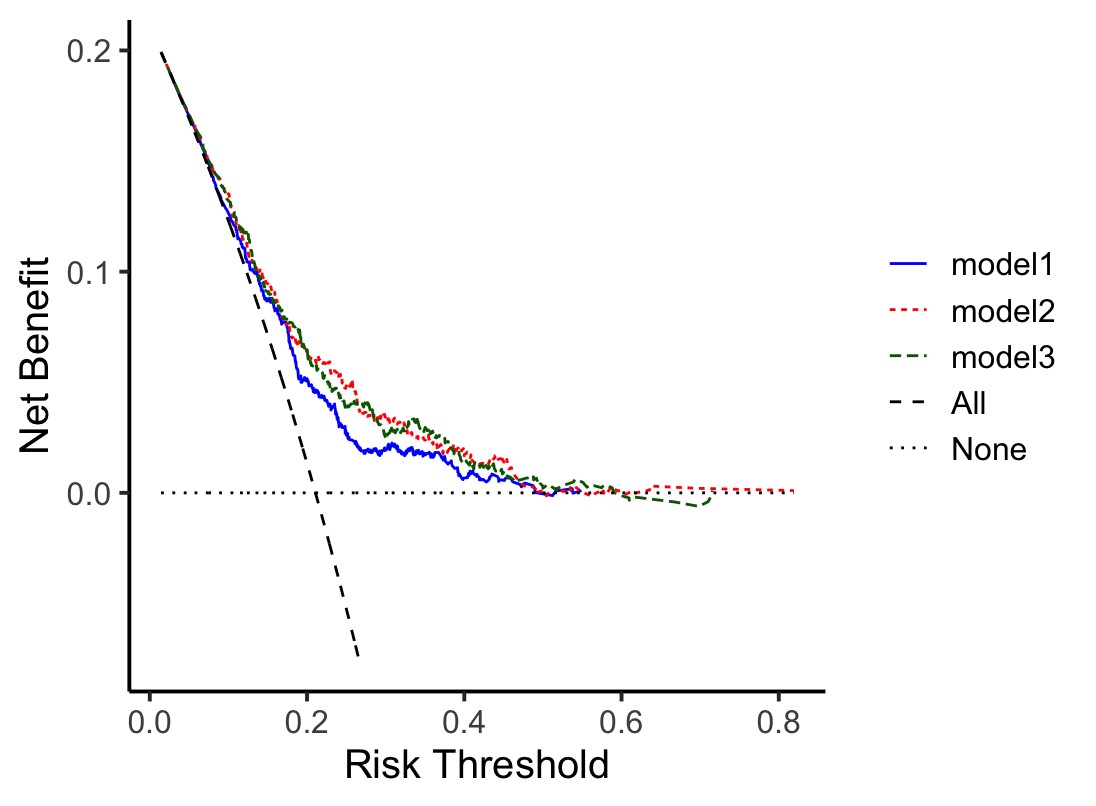


**Notes**: Model 1 is identical to the original Model 1, Model 2 corresponds to the original Model 3, and Model 3 replaces the categorized ACAG with its continuous form.

As is shown in the figure S1, the continuous specification yielded a lower clinical net benefit than the dichotomized version. Restricted cubic spline analysis further showed a marked upward inflection in the hazard ratio at the cut-off identified by the ROC-derived Youden index, suggesting that this threshold may offer clinically actionable guidance. Nevertheless, since the optimal ACAG cut-point has varied across studies that used different endpoints, larger datasets and additional outcome analyses are still required to fix a definitive threshold.

**Figure S2** Kaplan-Meier survival curves for clinical outcomes by ACAG quartiles.

| 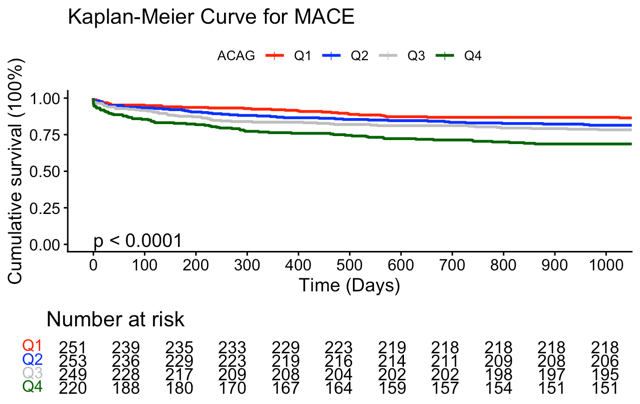 | 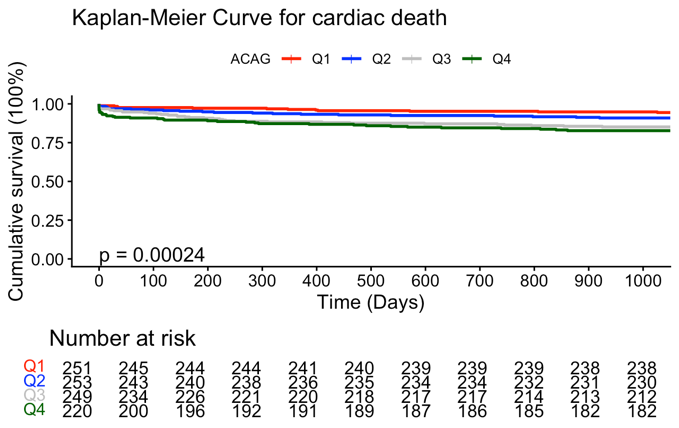 |
| --- | --- |
| 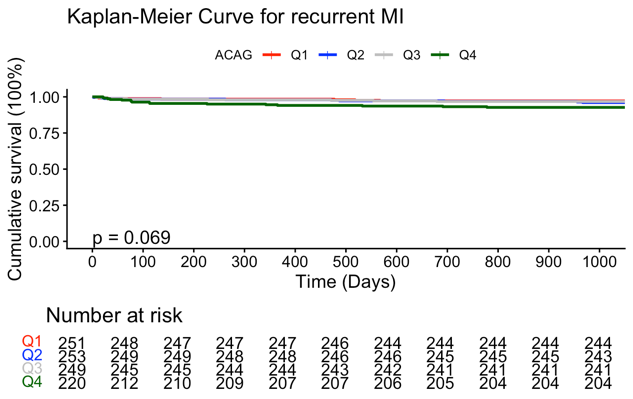 | 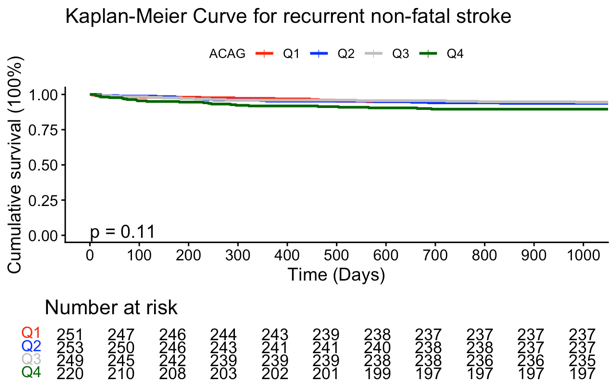 |
| 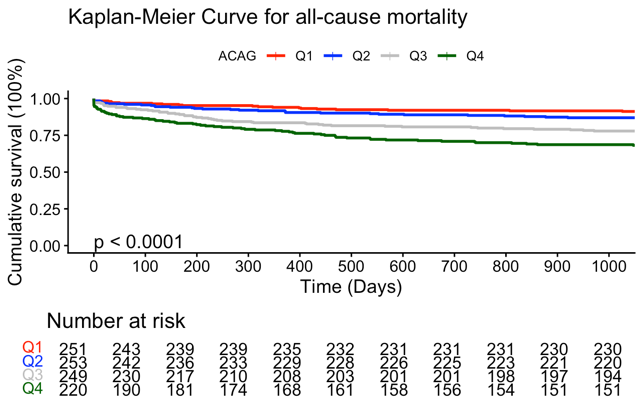 | 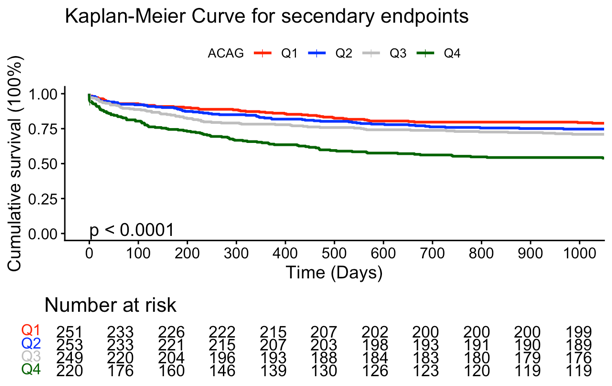 |

**Figure S3** Decision Curve Analysis (DCA) of ACAG quartiles for predicting MACEs.


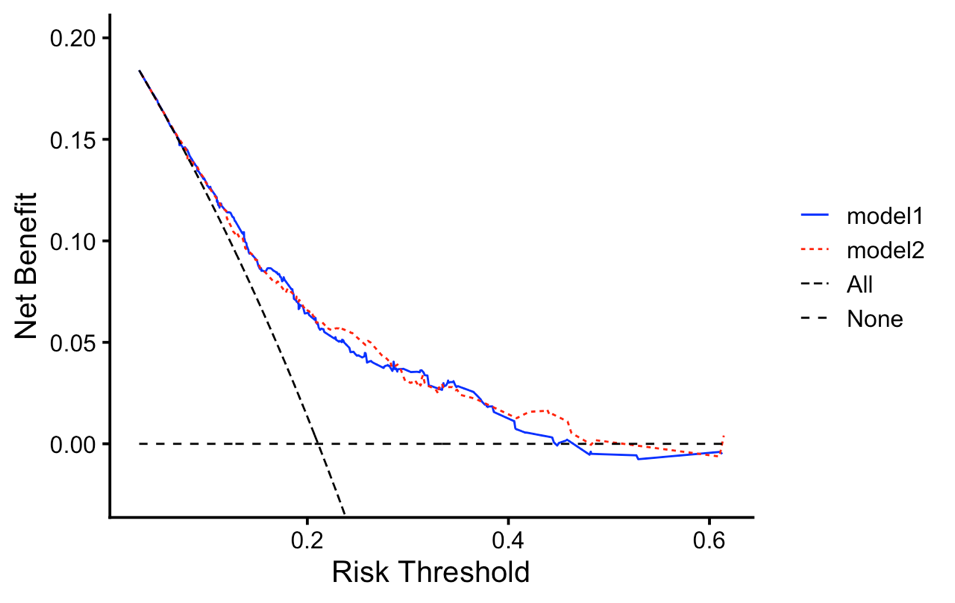


**Notes:** Model 1 was identical to the original Model 3 (ACAG: binary by cutoff), Model 2 used ACAG quartiles instead.

**Table S1** Cox Regression Analysis (grouped by quartiles)

**Table S2** Association of ACAG with MACEs by different grouping strategies.

**Table S3** Sensitivity analysis: ACAG-MACEs association by model specification

**Table S1** Cox Regression Analysis (grouped by quartiles)

| Multivariable Cox Regression | | | |
| --- | --- | --- | --- |
|  | | HR (95%CI) | P |
| Gender | | 1.079(0.806-1.444) | 0.609 |
| Age | | 1.033(1.018-1.048) | <0.001 |
| Diabetes mellitus | | 1.511(1.138-2.007) | 0.004 |
| eGFR | | 0.986(0.978-0.994) | 0.001 |
| ACAG | |  |  |
|  | Q1 | Ref |  |
|  | Q2 | 1.185(0.761-1.846) | 0.453 |
|  | Q3 | 1.385(0.898-2.135) | 0.140 |
|  | Q4 | 2.070(1.346-3.181) | 0.001 |
| **multi-vessel disease**  disease | | 2.525(1.102-5.787) | 0.029 |
| diuretics | | 1.653(1.195-2.286) | 0.002 |
| LVEF | | 0.987(0.975-0.999) | 0.033 |

**Abbreviations:** eGFR, estimated glomerular filtration rate; LVEF, left ventricular ejection fraction; Q1, 7.4<=ACAG<14.21; Q2, 14.21<=ACAG<16.15; Q3, 16.15<=ACAG<19.09; Q4, 19.09<=ACAG<35.68.

**Table S2** Association of ACAG with MACEs by different grouping strategies.

| **Group Strategy** | Patients  Events No ,% | HR (95%CI) | P |
| --- | --- | --- | --- |
| **Dichotomous** （grouped by cutoff value） |  |  |  |
| Q1(<17.44mmol/L) | 95 (15.78%) | Ref |  |
| Q2(≥17.44mmol/L) | 110 (29.65%) | 1.825 (1.367-2.436) | <.001 |
| **Quartiles** |  |  |  |
| Q1 (7.4≤ACAG<14.21)) | 32 (13.17%) | Ref |  |
| Q2 (14.21≤ACAG<16.15) | 47 (19.42%) | 1.185(0.761-1.846) | 0.453 |
| Q3 (16.15≤ACAG<19.09) | 50 (20.41%) | 1.385(0.898-2.135) | 0.140 |
| Q4 (19.09≤ACAG<35.68) | 76 (31.28%) | 2.070(1.346-3.181) | 0.001 |
| P for linear trend |  |  | 0.002 |
| **Continuous** (per 1-unit increase) |  | 1.252 (1.098-1.427) | <.001 |

**Table S3** Sensitivity analysis: ACAG-MACEs association by model specification

| **Group Strategy** | AIC | Bootstrap AIC Mean | Optimal Frequency (%) | BIC |
| --- | --- | --- | --- | --- |
| **Dichotomous** （grouped by cutoff value） | 2699.16 | 2683.10 | 86.3% | 2725.75 |
| **Quartiles** | 2706.18 | 2690.78 | 5.7% | 2739.41 |
| **Continuous** (per 1-unit increase) | 2702.83 | 2689.08 | 8.0% | 2729.41 |
